# Metabolomic and transcriptomic changes in mungbean (*Vigna radiata* (L.) R. Wilczek) sprouts under salinity stress

**DOI:** 10.3389/fpls.2022.1030677

**Published:** 2022-10-17

**Authors:** Insu Lim, Minseo Kang, Byeong Cheol Kim, Jungmin Ha

**Affiliations:** Department of Plant Science, Gangneung-Wonju National University, Gangneung, South Korea

**Keywords:** mungbean sprout, salinity stress, ultra-high-performance liquid chromatography, phenylpropanoid compound, RNA-seq, gene expression

## Abstract

Mungbean (*Vigna radiata*) sprouts are consumed globally as a healthy food with high nutritional values, having antioxidant and anticancer capacity. Under mild salinity stress, plants accumulate more secondary metabolites to alleviate oxidative stress. In this study, metabolomic and transcriptomic changes in mungbean sprouts were identified using a reference cultivar, sunhwa, to understand the regulatory mechanisms of secondary metabolites in response to salinity stress. Under salinity conditions, the contents of phenylpropanoid-derived metabolites, including catechin, chlorogenic acid, isovitexin, *p*-coumaric acid, syringic acid, ferulic acid, and vitexin, significantly increased. Through RNA sequencing, 728 differentially expressed genes (DEGs) were identified and 20 DEGs were detected in phenylpropanoid and flavonoid biosynthetic pathways. Among them, 11 DEGs encoding key enzymes involved in the biosynthesis of the secondary metabolites that increased after NaCl treatment were significantly upregulated, including dihydroflavonol 4-reductase (log_2_FC 1.46), caffeoyl-CoA O-methyltransferase (1.38), chalcone synthase (1.15), and chalcone isomerase (1.19). Transcription factor families, such as *MYB*, *WRKY*, and *bHLH*, were also identified as upregulated DEGs, which play a crucial role in stress responses in plants. Furthermore, this study showed that mild salinity stress can increase the contents of phenylpropanoids and flavonoids in mungbean sprouts through transcriptional regulation of the key enzymes involved in the biosynthetic pathways. Overall, these findings will provide valuable information for molecular breeders and scientists interested in improving the nutritional quality of sprout vegetables.

## Introduction

Mungbean (*Vigna radiata* (L.) R. Wilczek) is an important legume crop grown over 6 million hectares worldwide (about 8.5% of the global pulse cultivation area) (Gayacharan et al., 2020). Mature mungbean seeds are valuable food sources of protein and starch in many developing countries in Asia (Nair et al., 2013). Mungbean sprouts have long been consumed globally because of their excellent texture and abundant nutrients, including ascorbic acid, dietary fiber, essential amino acids, and vitamins (Gan et al., 2016; Ebert et al., 2017; Singh et al., 2017). In particular, six polyphenolic compounds, including caffeic acid, ferulic acid, gallic acid, *p*-coumaric acid, catechin, and rutin, have been identified in mungbean sprouts (Gan et al., 2016), which help maintain human health and prevent chronic diseases (Kim et al., 2012; Nderitu et al., 2013; Pudenz et al., 2014; Stagos, 2020). Owing to these nutritional values, mungbean sprout consumption has increased, and the quality of sprout vegetables has attracted consumers’ attention globally.

Plants accumulate polyphenolic compounds as defense metabolites against harmful environments, and this accumulation varies depending on different stress exposures, such as high/low temperature, salinity, ultraviolet, and drought (Ksouri et al., 2008; Yang et al., 2018; Sharma et al., 2019; Chrysargyris et al., 2020). Under salinity conditions, osmotic, ionic, and oxidative stresses affect most plants, inducing morphological and biochemical changes (Yang and Guo, 2018). While severe salinity disrupts general plant development through ionic toxicity and water imbalance (Yang and Guo, 2018), mild NaCl treatment can considerably enhance the polyphenol contents and antioxidant activity in many plant species, such as rice seedlings as well as mungbean, broccoli, buckwheat, and radish sprouts (Yuan et al., 2010; Lim et al., 2012; Guo et al., 2014; Koodkaew, 2019). Similarly, many metabolic studies have shown that anthocyanin and flavonoid metabolisms are significantly influenced by mild NaCl treatment, followed by enhanced production of catechin, chlorogenic acid, ferulic acid, *p*-coumaric acid, syringic acid, and vitexin (Lim et al., 2012; Hassini et al., 2017; Sarker and Oba, 2018; Banik and Bhattacharjee, 2020; Benincasa et al., 2021). Therefore, salinity stress can play a crucial role in regulating the quality of the nutritional values of plant ingredients.

With RNA-seq-based transcriptome analysis, the gene expression patterns and regulation network in response to salinity have been widely investigated in several plants, including cotton (Wang et al., 2012), rice (Chandran et al., 2019), buckwheat (Ma et al., 2019), soybean (Fan et al., 2013), and medicine plants (Ben Abdallah et al., 2016). The expression of the genes involved in the biosynthetic pathways of flavonoids and phenylpropanoids can be increased by NaCl treatment in *solanum nigrum*, including phenylalanine ammonialyase, chalcone synthase, and flavonol synthase (Ben Abdallah et al., 2016). Furthermore, various transcription factor (TF) families play a crucial role in salinity stress responses in plants, such as NACs, MYBs, WRKYs, and bHLHs (Eulgem and Somssich, 2007). Overexpression of TaNAC29, AtMYB20, FtMYB10, and OsMYB3R-2 enhanced salt tolerance in transgenic Arabidopsis (Dai et al., 2007, 3; Cui et al., 2013, 3; Xu et al., 2015; Gao et al., 2016). TaMYB56-B, MdSIMYB1, and OsNAC6 were associated with salinity stress responses in wheat, apple, and rice, respectively (Nakashima et al., 2007; Shen et al., 2017).

During the mungbean germination stage, polyphenol accumulation is affected by growth conditions and stresses, such as temperature, salinity, and drought (Kim et al., 2009b). When mungbean sprouts are exposed to NaCl, the total phenolic and flavonoid contents increase, resulting in enhanced antioxidant activities (Koodkaew, 2019). However, only a few transcriptome studies have explored resistance to fungi and drought stress in mungbean seeds/leaves (Kumar et al., 2020), and little is known about the genetic regulation of secondary metabolites for abiotic stresses in mungbean sprouts.

This study’s aim is to investigate the effects of NaCl treatment on morphological, metabolomic, and transcriptomic changes in mungbean sprouts using two cultivars, dahyeon and sunhwa. Dahyeon has been reported to accumulate relatively higher polyphenols in its sprouts compared to other cultivars and sunhwa is a mungbean reference cultivar (Kim et al., 2009a; Habibzadeh and Yagoob, 2014; Kang et al., 2014; Yoseph Ganta et al., 2021). We qualified and quantified the polyphenolic compounds in mungbean sprouts after NaCl treatment and identified the key enzymes involved in the biosynthetic pathways of these compounds. Our findings provide further insights into the molecular mechanisms associated with salinity stress and can help improve the nutritional quality and morphological characteristics of sprout vegetables through genetic engineering in legume crops.

## Materials and methods

### Plant materials and NaCl treatment

Two mungbean cultivars, dahyeon and sunhwa (VC1973A), were used in our experiments. Seeds were harvested at the Gangneung-Wonju National University Experimental Farm in Gangneung, South Korea (37.77°N, 128.86°E). The seeds were soaked in distilled water for 17 h at 37°C using an incubator (Jeiotech, ISS-4075R, Korea) for germination. The germinated seeds were transplanted into a plant growth chamber (Sundotcom, ST001A, Korea) and cultivated for three days at 28 ± 2°C in the dark. Water spraying automatically occurred for 4 min every 2 h (Kim et al., 2021). During cultivation, mungbean sprouts were soaked in different concentrations (0, 50, 100, and 200 mM) of NaCl solution for 30 min every 12 h (KisanBio, MB-S4636, Korea). Mungbean sprouts were harvested on the third day after germination and stored at −70°C for RNA extraction (Lim et al., 2022). Fifty seeds were cultured for each experimental group, 30 sprouts were randomly picked for further analysis, and five sprouts were used for each biological replication of RNA extraction. The lengths of the root and hypocotyl and the thickness of the hypocotyl were measured using ImageJ (Schneider et al., 2012).

### Extraction procedure

Mungbean sprouts were fully dried at 70°C for 24 h using an incubator (Gan et al., 2017). The sample was ground into a fine powder using mortar and pestle and 0.05 g powder of each sample was extracted using 70% ethanol (w/v, 1:10) for 24 h in the dark. Mixtures were vortexed and sonicated for 15 min and centrifuged at 13,000 rpm for 10 min. The supernatant was filtered through syringe filters (SMART I LAB, SPF0213-1, USA, 0.22 um) and diluted with 70% ethanol for further analysis.

### Determination of total phenol content

The contents of total phenol were measured using Folin-Ciocalteu colorimetric method with slight modification (Singleton and Rossi, 1965). Gallic acid (0, 50, 100, and 200 mg/L) was used as a standard for quantification. 50 µL Folin–Ciocalteu (Sigma-Aldrich^®^, 47641, USA) was added to each mungbean sprout extraction (100 µL, 10,000 ppm) in an EP tube. After 5 min, 300 µL of 20% Na_2_CO_3_ (FUJIFILM, 199-01605, Japan) solution was added. After 15 min in dark condition, distilled water was added up to 1 mL. After 2 min of centrifuge, 200 ul of the supernatants were transferred to a 96-well plate, and absorbance was measured at 740 nm wavelength using a spectrophotometer (Thermo Scientific MIB, Multiskan FC, Korea, 738 nm), with three replications. The results were expressed as milligrams gallic acid equivalent (GAE) per 1 g of dry weight (GAE mg/g).

### Determination of total flavonoid content

The contents of total flavonoids were measured using the aluminum nitrate colorimetric method with slight modifications (Zhishen et al., 1999). Quercetin (Sigma-Aldrich^®^, Q4951, USA) (0, 50, 100, and 200 mg/L) was used as standard. The mungbean sprouts extract (500 µL, 50,000 ppm) was mixed with a 10% aluminum nitrate (100 µL) solution and 1 M potassium acetate solution (100 µL). After 40 min in dark conditions and 2 min of centrifuge, 150 µL of the supernatants were transferred to a 96-well plate, and absorbance was measured at 429 nm wavelength using a spectrophotometer, with three replications. The results were expressed as milligrams quercetin equivalent (QAE) per 1 g of dry weight (QAE mg/g).

### Determination of antioxidant activity through DPPH and ABTS assays

DPPH radical scavenging activity was measured using OxiTec™ DPPH Antioxidant Assay Kit (BIOMAX, BO-DPH-500, Korea) according to the manufacturer’s protocol. Trolox (0, 40, 60, 80, and 100 mg/L) was used as the standard. Each extraction (20 µL, 50,000 ppm) was mixed with assay buffer (80 µL) and DPPH radical (100 µL) in 96-well plates, with three replications. The samples were preserved in the dark for 30 min, and absorbance was measured at 520 nm using a spectrophotometer. The result was expressed as a percentage of scavenging achieved by the DPPH.

ABTS radical scavenging activity was measured as described by Yen (Yen and Chen, 1995). Ascorbic acid (FUJIFILM, 012–04802, Japan) (0, 1, 5, 10, 25, 50, and 100 mg/L) was used as the standard. ABTS (Roche, Cat. No. 10102946001, Swiss) 7.4 mM solution and 2.6 mM potassium persulfate (YAKURI, 28718, Korea) were diluted to a 1:1 ratio (v/v). ABTS solution (180 μL) was added to each extraction (20 µL, 1,000 ppm) in 96-well plates, with three replications. The samples were kept in dark for 10 min, their absorbance was measured at 720 nm using a spectrophotometer. The result was expressed as a percentage of scavenging achieved by the ABTS.

### Ultra-high-performance liquid chromatography analysis

Ultra-high-performance liquid chromatography (UHPLC) analysis was conducted using the Shimadzu UHPLC system (Nexera series equipped with MPM-40, SCL-40, SPD-M40, LC-40, SIL-40, and CTO-40 unit from Shimadzu, Kyoto, Japan) with a UV-vis detector. Separation was conducted using a C18 column (Shimadzu, Kyoto, Japan; 250 × 4.6 mm, 5 µm). Ultrapure water (Thermo Fisher, W5-4, Korea) with 0.1% ortho-phthalaldehyde buffer and acetonitrile (Thermo Fisher, 022927 M6, Korea) were used as solvents A and B, respectively. The flow rate was 0.2 mL/min and the column temperature was set at 40°C. The ratio of solvent B proceeded with 5%, increased to 30% for 0–8 min, increased to 36% for 8–20 min, and decreased to 5% for 20–25 min. 14 compounds were used as standard compounds: caffeic acid (CFN99190), catechin (CFN99646), chlorogenic acid (CFN99116), gallic acid (CFN99624), isovitexin (CFN98620), kaempferol (CFN98838), myricetin (CFN98877), neochlorogenic acid (CFN97472), *p*-coumaric acid (CFN97218), quercetin (CFN99272), resveratrol (CFN98791), syringic acid (CFN98884), t-ferulic acid (CFN99158), and vitexin (CFN98601). The standard compounds were purchased from ChemFaces (Wuhan, China). The results were obtained based on standard calibration curves (10-100 ppm) with a tri-repeat and expressed as milligrams per 100 g of dry weight (mg/100g).

### cDNA library construction and transcriptome sequencing

Total RNA was isolated from whole mungbean sprouts using a Ribospin™ Plant RNA extraction Kit (GeneAll, Songpa-gu, South Korea) according to the manufacturer’s protocol. The cDNA libraries for RNA-seq were constructed using a TruSeq Stranded mRNA LT Sample Prep Kit (Illumina, Inc., San Diego, CA, USA). Six libraries were constructed from the treatment and control groups, with three biological replications in each group. The size and quality of the libraries used for sequencing were checked using a 2100 Bioanalyzer (Agilent Technologies Inc., Santa Clara, CA, USA). Sequencing runs were conducted in paired-end mode using a Truseq SBS Kit on the Illumina NovaSeq 6000 platform. RNA sequencing data were deposited in the NCBI SRA database (PRJNA853140).

### Identification and analysis of differentially expressed genes

Raw reads were filtered out (q-value > 20) using trimmomatic version 0.36 (http://www.usadellab.org/) with default parameters (Bolger et al., 2014). The reference genome and gene annotation data were downloaded from the Seoul National University Crop Genomics Lab (http://plantgenomics.snu.ac.kr) (Ha et al., 2021). The filtered reads were mapped to the mungbean reference genome using Tophat2 (Kim et al., 2013, 2). Counts per million mapped read values were calculated for 30,999 genes using FeatureCounts (Liao et al., 2014). DEGs were counted using Edge-R (Robinson et al., 2010), which were defined as genes with an absolute log_2_ fold change (FC) value greater or equal to 1 between the two groups (control over treatment).

### Functional annotation of DEGs

Gene ontology (GO) enrichment analysis was performed using BINGO software (http://www.psb.ugent.be/cbd/papers/BiNGO/Home.html) (Maere et al., 2005). The Kyoto Encyclopedia of Genes and Genomes (KEGG) ontology (KO) pathway enrichment analysis was conducted using DAVID (https://david.ncifcrf.gov/) (Sherman et al., 2022). The TFs were identified using PlantTFDB (http://planttfdb.gao-lab.org/) (Tian et al., 2020).

### Validation by real-time quantitative reverse transcription PCR

Real-time quantitative reverse transcription PCR (qRT-PCR) was performed using a PrimeScript RT reagent Kit with gDNA Eraser (TaKaRa Bio Inc., San Jose, CA, USA) according to the manufacturer’s protocol. Thirty cycles of PCR amplification were conducted using gene-specific primers. Cytochrome P450 family 20 (CYP20) was used as an internal control gene. The primer sequences are listed in the supplementary information (**Table S3**).

### Statistical analysis

Statistical analysis was performed in R (R Core Team, 2017). The significant differences were calculated by a one-way analysis of variance followed by Duncan’s multiple test; *p*-value <0.05 was considered statistically significant.

## Results

### Development of mungbean sprouts under NaCl treatments

**Figure 1:**
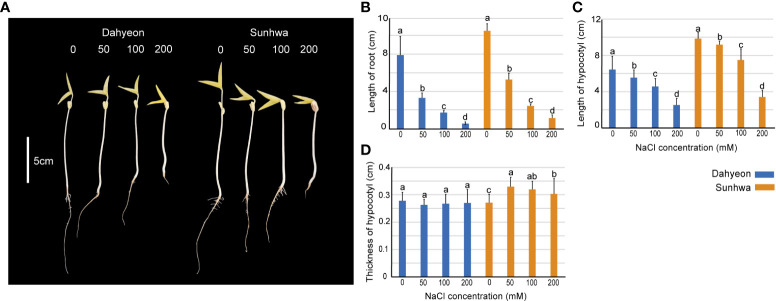
Morphological changes in mungbean sprouts of dahyeon and sunhwa cultivars under different concentrations of NaCl treatment (0, 50, 100, and 200 mM). **(A)** Picture of sprouts, **(B)** length of root, **(C)** length of hypocotyl, and **(D)** thickness of hypocotyl. The color bar indicates the mungbean cultivars, blue represents dahyeon and orange represents sunhwa. The results were expressed with a standard error bar, and different lowercase letters indicate statistical significance (*P* < 0.05).

We first assessed the effect of NaCl treatment on the development of mungbean sprouts using a gradient of different NaCl concentrations (0, 50, 100, and 200 mM) (**Figure 1A**). The length and thickness of hypocotyl and the length of the roots were measured in dahyeon and sunhwa (**Figure 1B**). The length of the roots and hypocotyl significantly decreased in both cultivars when the NaCl concentration increased (**Figures 1B, C**). While hypocotyl thickness showed no significance in dahyeon, it increased significantly in sunhwa under NaCl treatments (**Figure 1D**).

### Dynamic changes in total phenol and flavonoid contents, and antioxidant activities under NaCl treatment

**Figure 2:**
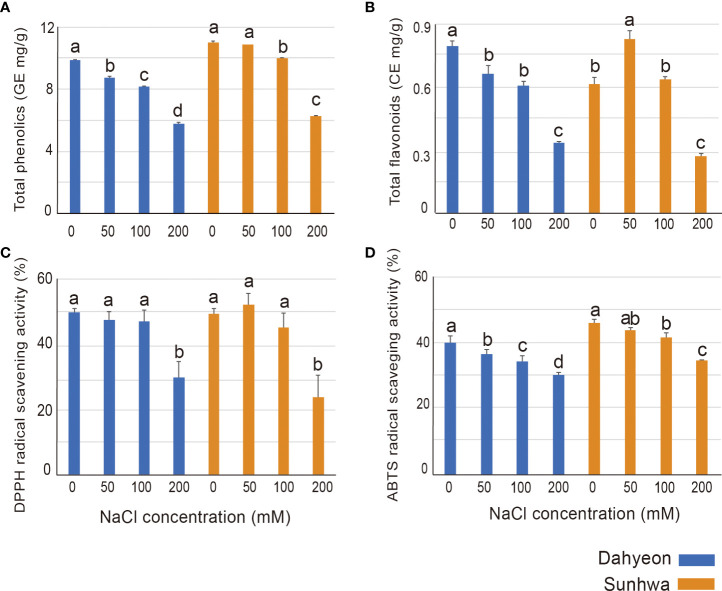
Comparison of **(A)** total phenolic contents, **(B)** total flavonoid contents, **(C)** antioxidant scavenging activity of DPPH, and **(D)** antioxidant scavenging activity of ABTS in the two mungbean cultivars cultivated on different concentrations of NaCl treatment (0, 50, 100, and 200 mM). The color bar indicates the mungbean cultivars, blue represents dahyeon and orange represents sunhwa. The results were expressed with a standard error bar, and different lowercase letters indicate statistical significance (*P* < 0.05).

We also evaluated the total phenol and flavonoid contents and antioxidant activity under different NaCl concentrations (**Figure 2**). In general, for both cultivars, the phenol contents decreased as the NaCl concentration increased (**Figure 2A**). However, for total flavonoid content, the two cultivars had opposite responses under low concentrations of NaCl treatment (50 mM, Na50). The flavonoid contents of dahyeon showed a significant decrease when NaCl concentrations increased, while those of sunhwa peaked under Na50 and then decreased under higher concentrations of NaCl (100 and 200 mM) (**Figure 2B**).

DPPH and ABTS assays were conducted to evaluate the antioxidant activity of the mungbean sprouts (**Figures 2C, D**). In the DPPH assay, no difference in the antioxidant activities was observed in both cultivars treated with 0, 50, and 100 mM NaCl, and the antioxidant activity significantly declined under 200 mM NaCl treatment (**Figure 2C**). The antioxidant activity detected through the ABTS assay was gradually reduced in both cultivars as NaCl concentration increased (**Figure 2D**).

### Profile of polyphenolic compounds using UHPLC analysis

Metabolic profiling was conducted on sunhwa sprouts treated with Na50, where flavonoid contents significantly increased (**Table 1**). To better understand the effects of salinity on polyphenolic compounds, we quantified and qualified 14 compounds in mungbean sprouts, including six flavonoids (catechin, isovitexin, kaempferol, myricetin, quercetin, and vitexin) and eight phenolic acids (caffeic acid, chlorogenic acid gallic acid, *p*-coumaric acid, neochlorogenic acid, resveratrol, syringic acid, and t-ferulic acid) using UHPLC.

**Table 1:** The effects of 50mM of NaCl treatment on the polyphenol composition of mungbean sprouts of sunhwa cultivar.

Chemical group	Compound	Content (mg/100g)		
		Control	NaCl treatment(50 mM)	Log_2_FC
Flavonoid	Catechin	114.2±6.8^b^	160.4±1.7^a^	
	Isovitexin	16.6±0.1^b^	25.8±0.4^a^	
	Kaempferol	ND	ND	
	Myricetin	328.6±2.1^a^	233.9±0.9^b^	
	Quercetin	132.8±1.1^a^	113.6±0.3^b^	
	Vitexin	75.5±0.4^b^	123.8±1^a^	
Phenolic acid	Caffeic acid	57.6±1.6 ^ns^	57.4±0.2 ^ns^	
	Chlorogenic acid	1144.1±5.7^b^	1444.2±7.1^a^	
	Gallic acid	143.3±2.2 ^ns^	146.8±0.7 ^ns^	
	Neochlorogenic acid	35.7±0.3^a^	30.4±0.9^b^	
	*p-*Coumaric acid	16.4±0.7^b^	26.2±0.1^a^	
	Resveratrol	15.6±0.1^a^	14.6±0.2^b^	
	Syringic acid	4.1±0.5^b^	6.5±1^a^	
	t-Ferulic acid	13.4±0.2^b^	18.1±0.2^a^	

The color box indicates log2-fold change values of each metabolite (Na 50 over control), blue and red represent lower and higher values, respectively. Different lowercase letters indicate statistical significance (P < 0.05). ND and NS mean non-detected and non-significance, respectively.
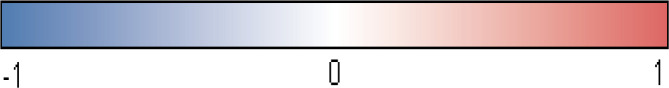

Among the 14 compounds, 13 compounds excluding kaempferol were detected in both the control and NaCl treatment samples (**Table 1**). Chlorogenic acid was the most abundant in both the control and NaCl treatments, with values of 1144.1 mg/100g and 1444.2 mg/100g, respectively (**Table 1**). Myricetin was the second most abundant compound, with values of 328.6 mg/100g (control) and 233.9 mg/100g (Na50), followed by gallic acid (143.3 and 146.8 mg/g), catechin (114.2 and 160.4 mg/100g), quercetin (132.8 and 113.6 mg/100g), and vitexin (75.5 and 123.8 mg/100g), respectively (**Table 1**).

The contents of seven compounds (catechin, chlorogenic acid, glycerin, isovitexin, *p*-coumaric acid, syringic acid, t-ferulic acid, and vitexin) were significantly increased after Na50 treatment (**Table 1**). In contrast, the contents of four compounds (myricetin, neochlorogenic acid, quercetin, and resveratrol) were significantly reduced under NaCl treatment (**Table 1**). In the other two compounds (caffeic acid and gallic acid), no significant differences were observed in their contents between the control and Na50 (**Table 1**).

### Transcriptome analysis

**Figure 3:**
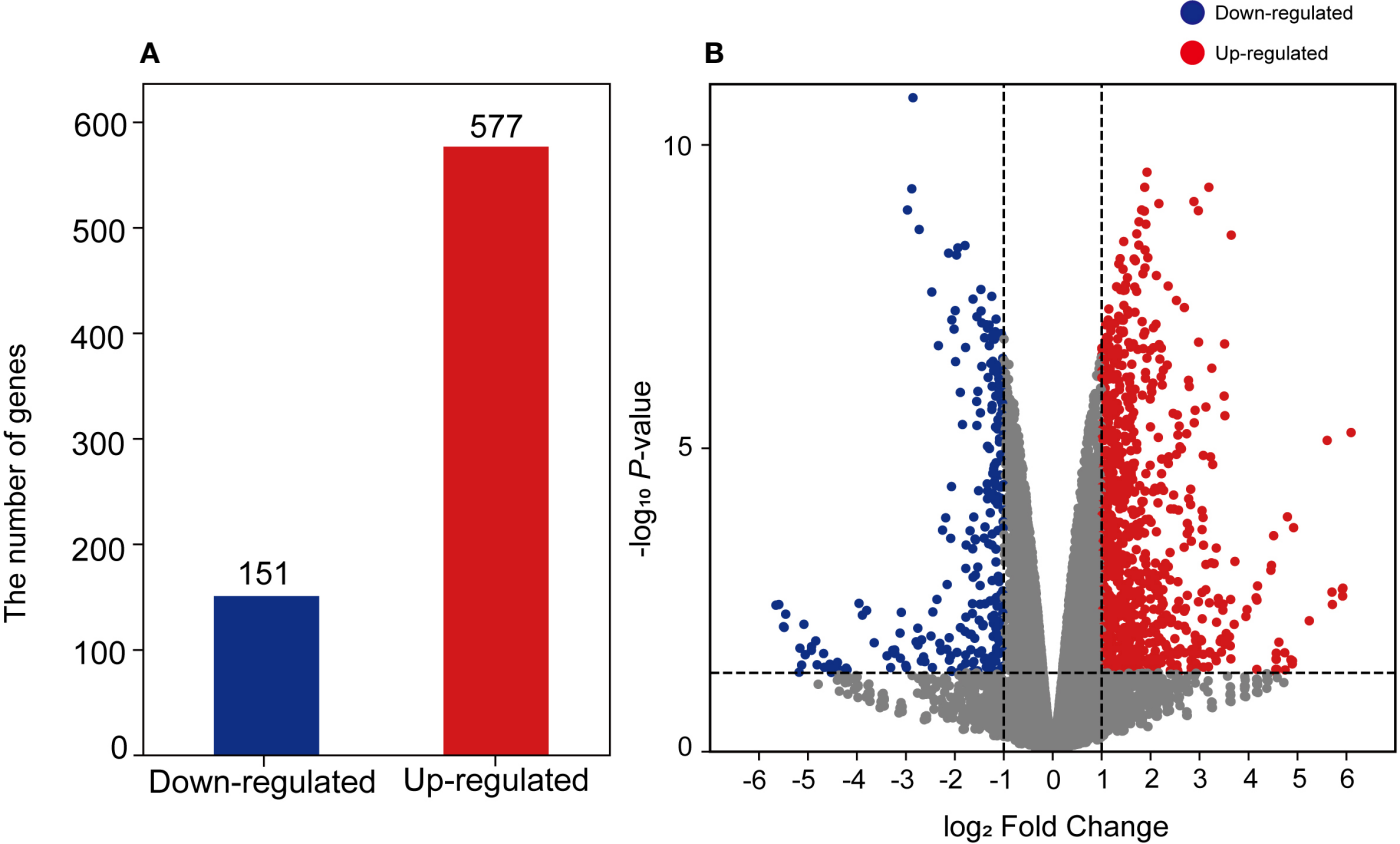
Visualization of identified DEGs using **(A)** bar plot and **(B)** volcano plot. The y-axis of the volcano plot indicates log2-fold change values of each DEG, and the x-axis indicates –log10 p-value. The color bar indicates gene regulation, blue represents downregulated genes and red represents upregulated genes.

To investigate the effects of salinity stress on transcriptional changes, we conducted RNA sequencing on sunhwa sprouts treated with Na50. In total, 96.8 Gb of filtered reads (16.1 Gb per library, on average) were obtained from the control and Na50 samples, with three replications (**Table S1**). On average, 98.08% of the reads were mapped properly against the mungbean reference genome (Ha et al., 2021) (**Table S1**). DEGs were identified between the NaCl treatment and control groups. A total of 728 DEGs were identified, and 151 and 577 genes were downregulated and upregulated after NaCl treatment, respectively (**Figures 3A, B**).

### Functional annotation of DEGs through enrichment analysis

**Figure 4:**
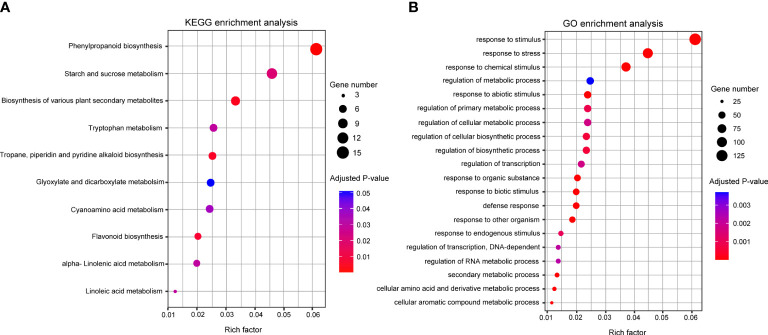
**(A)** KEGG and **(B)** GO pathway enrichment analyses of DEGs. The y-axis indicates the pathway or term name, and the x-axis indicates the enriched factor in each pathway and term. The circle size indicates the number of genes. The color bar indicates the adjusted p-value, blue and red represent higher and lower values, respectively. GO, Gene Ontology; KEGG, Kyoto Encyclopedia of Genes and Genomes.

**Figure 5:**
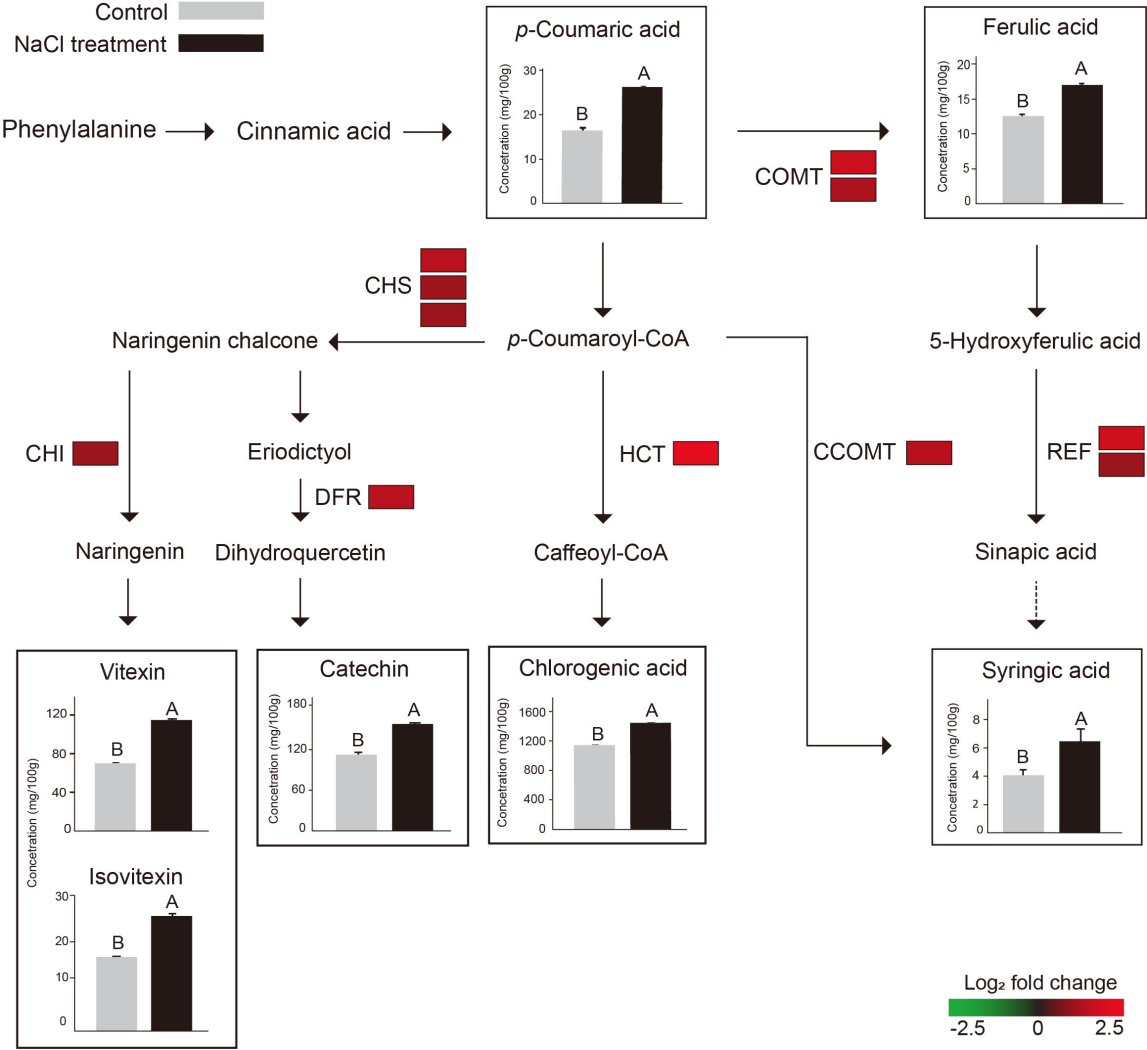
Schematic illustration of phenylpropanoid pathway with the expression levels of 11 DEGs (COMT, CCOMT, CHS, CHI, HCT, REF1, and DFR) and the contents of 7 metabolites that increased after NaCl treatment (catechin, chlorogenic acid, glycerin, isovitexin, *p*-coumaric acid, syringic acid, t-ferulic acid, and vitexin). The color scale indicates the log2-fold change (FC) value of each DEG, green and red represent lower and higher expression levels, respectively. The color blocks indicate FC of expression levels of paralogs of each DEG. The bar plots in the square show the comparison of metabolite contents between the control (gray) and NaCl treatment (black). The different uppercase letters indicate statistical significance (*P* < 0.05). The dotted line indicates an enzymic reaction that has not been fully identified in plants. COMT, catechol O-methyltransferase; CCOMT, caffeoyl-CoA O-methyltransferase; CHS, chalcone synthase; CHI, chalcone isomerase; HCT, shikimate O-hydroxycinnamoyl transferase; REF1, coniferyl-aldehyde dehydrogenase; DFR, Dihydroflavonol 4-reductase;.

The KEGG pathway and GO enrichment analyses were conducted to understand DEG’s molecular functions. In KEGG pathway analysis, 133 DEGs were assigned to 11 pathways and the top three pathways were “phenylpropanoid biosynthesis” (15), “starch and sucrose metabolisms” (11), and “biosynthesis of various plant secondary metabolites” (8) (**Figure 4A**). In GO analysis, 464 DEGs were clustered in 104 categories, and the major clusters were “response to stimulus” (137), followed by “response to stress” (100) and “response to chemical stimulus” (83) (**Figure 4B**). Within the categories involved in secondary metabolites, “phenylpropanoid metabolic process” (18) and “flavonoid metabolic process” (6) were identified as enriched categories. Based on the enrichment analyses, 46 DEGs were identified as genes related to the biosynthesis of phenylpropanoid metabolites. The 46 DEGs were mapped into the phenylpropanoid pathway in KEGG, and 11 DEGs were selected as key genes encoding the enzymes directly involved in the biosynthesis of the secondary metabolites that increased significantly after NaCl treatment (**Figure 5**; **Table S2**).

**Figure 6:**
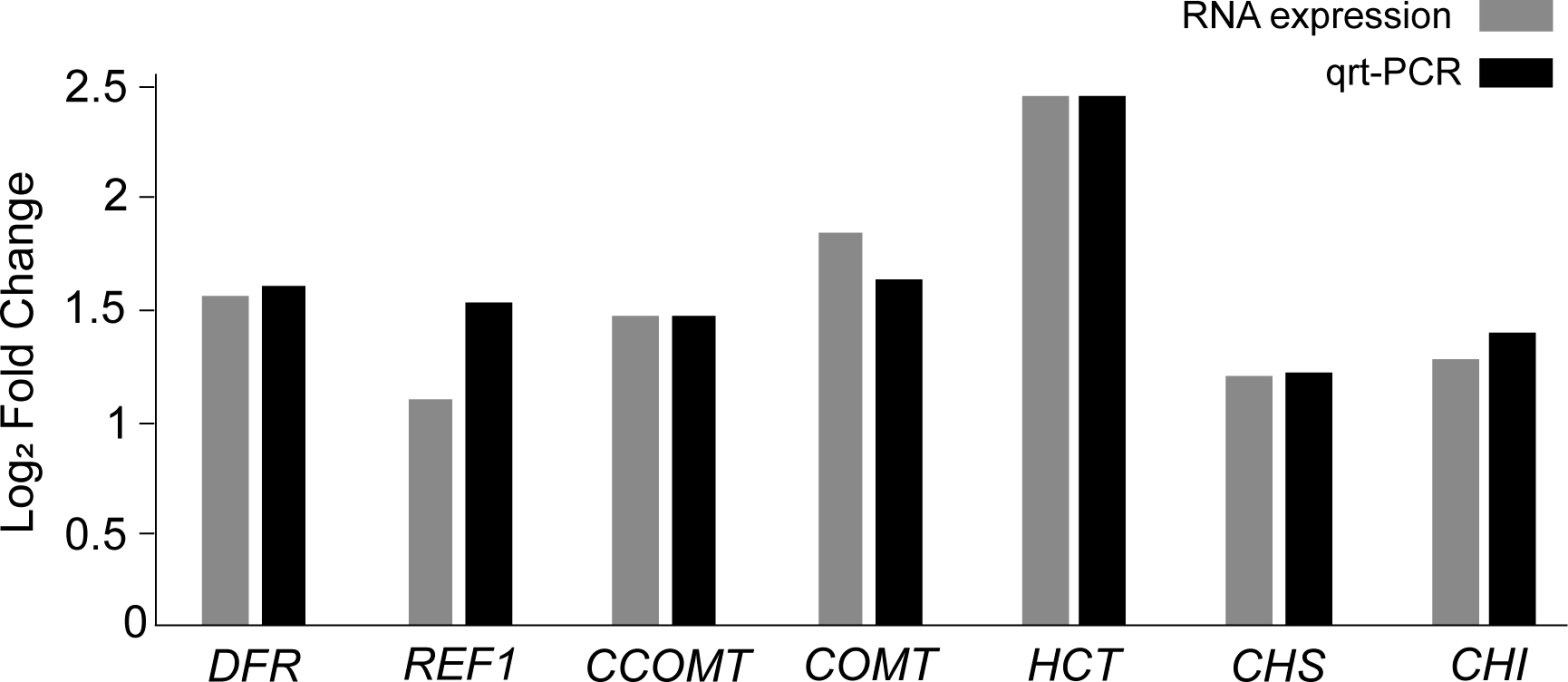
Validation of the RNA expression of DEGs by qRT-PCR analysis.

The 11 DEGs encode seven enzymes: catechol O-methyltransferase (COMT: Vradi03g00001673.1, Vradi02g00004009.1), caffeoyl-CoA O-methyltransferase (CCOMT: Vradi09g00001754.1), chalcone synthase (CHS: Vradi09g00003301.1, Vradi09g00003302.1, Vradi09g00003303.1), chalcone isomerase (CHI: Vradi03g00001100.1), shikimate O-hydroxycinnamoyl transferase (HCT: Vradi09g00002363.1), coniferyl-aldehyde dehydrogenase (REF1: Vradi09g00002363.1, Vradi02g00003639.1), and dihydroflavonol 4-reductase (DFR: Vradi07g00001339.1) (**Table S3** and **Figure 5**). In addition, the 11 DEGs were significantly upregulated under salinity conditions, and their Log_2_ FC values were COMT (1.75, 1.31), CCOMT (1.38), CHI (1.18), CHS (1.14, 1.13, 1.1), HCT (2.35), REF1 (1.85, 1), and DFR (1.46) (**Figure 5**). For validation of RNA-seq data, we conducted qRT-PCR and found that the result of qRT-PCR well agree with that of RNA-seq, indicating the reliability of the RNA-seq data (**Figure 6**).

## Discussion

Mungbean sprouts are a good source of natural antioxidants, including catechin, chlorogenic acid, vitexin, isovitexin, and *p*-coumaric acid (Kim et al., 2009b; Guo et al., 2012; Gan et al., 2016; Singh et al., 2017). These secondary metabolites provide various health benefits for the human body, such as anticancer, antioxidant, antimicrobial, anti-inflammatory, and antitumor effects (Gorzynik-Debicka et al., 2018; Stagos, 2020; Teodor et al., 2020). In plants, abiotic stresses, such as salinity conditions, significantly influence secondary metabolite accumulation (Ksouri et al., 2008; Yang et al., 2018; Sharma et al., 2019; Chrysargyris et al., 2020). Many studies have demonstrated that mild NaCl treatment can improve secondary metabolite contents in various vegetables (Lim et al., 2012; Hassini et al., 2017; Sarker and Oba, 2018; Banik and Bhattacharjee, 2020; Benincasa et al., 2021). In the present study, we investigated the interactive effects of salinity stress on transcriptomic and metabolomic changes in mungbean sprouts.

NaCl treatment significantly suppressed mungbean sprout development (**Figure 1**). The root and hypocotyl length was reduced in both cultivars as NaCl concentration increased from 50 to 200 mM (**Figures 2A, B**). These results agree with previous findings that osmotic imbalance caused by salinity results in a significant reduction in root and hypocotyl length in maize (Khodarahmpour et al., 2012), wheat (Akbari et al., 2007), cowpea (Dantas et al., 2005), weed species (Hakim et al., 2011), tomato (Abdel-Farid et al., 2020), and mungbean (Saha et al., 2010). This inhibition of root growth by salinity stress is obviously a drawback in plants (Yin et al., 2022). However, in bean sprout production, long roots are considered a totally undesirable feature to reduce sprout texture, whereas a certain length and thickness of hypocotyl are required (Price, 1988). A previous study suggests that the most suitable features of mungbean sprouts are short roots and hypocotyl longer and thicker than 5 cm in length and 2 mm in diameter (Buescher and Chang, 1982). In our observation, sunhwa treated with Na50 showed an increase in hypocotyl thickness from 2.7 to 3.3 mm, maintaining a hypocotyl length over 8 cm (**Figures 1B, C**), indicating that mild NaCl treatment can promote the morphological quality of mungbean sprouts in consumers’ favor.

The total flavonoid contents increased in sunhwa at Na50 (**Figure 2B**), which corroborates previous findings that NaCl treatment can enhance the total flavonoid contents in wheat (Kiani et al., 2021), tomato (Abdel-Farid et al., 2020), and rapeseed sprout (Falcinelli et al., 2017). However, the total flavonoid contents gradually decreased in dahyeon as NaCl concentration increased (**Figure 3B**). This opposite response might be caused by sensitivity/insensitivity of the two cultivars to salt stress. Sunhwa (VC1973A) is a reference mungbean cultivar developed by the Asian Vegetable Research and Development Center ensuring high yield and insensitivity against diverse stresses (Habibzadeh and Yagoob, 2014; Kang et al., 2014; Yoseph Ganta et al., 2021). Secondary metabolites can be differentially regulated in salt-tolerant or sensitive cultivars under salinity condition (Borghesi et al., 2011; Linić et al., 2019). In the Brassicaceae family, the contents of phenolic acid were higher in more salt-tolerant varieties, such as white cabbage and kale, than in salt-sensitive varieties (Linić et al., 2019). In tomatoes, the accumulation of total anthocyanin contents differed depending on genotypes under NaCl treatment (Borghesi et al., 2011). Our results, along with those from other studies, reveal that the genotype with high salinity tolerance might be a valuable breeding material for high contents of antioxidant compounds in mungbean sprouts.

In this study, the antioxidant activity of mungbean sprouts was measured using two colorimetric methods (**Figures 2C, D**). DPPH and ABTS are both widely used for measuring the antioxidant activity of plant extraction (Floegel et al., 2011). While ABTS assay is suitable to measure both hydrophilic and lipophilic antioxidants, DPPH assay is more suited to measure hydrophobic systems (Floegel et al., 2011). We found that the antioxidant activities obtained by ABTS and DPPH assays presented slightly different trends (**Figures 2C, D**). In the ABTS assay, the antioxidant activity gradually declined as NaCl concentration increased, whereas no significant differences were observed in the DPPH assay under mild concentrations of NaCl (**Figures 2C, D**). This difference between the two assays indicates that the antioxidants mainly affected by mild NaCl treatment (0-100 mM) might be hydrophilic phytochemicals in mungbean sprouts.

Although a few studies have reported the effects of NaCl treatment on the total phenol and flavonoid contents in mungbean sprouts, the changes in the contents of individual phytochemicals along with transcriptomic changes remain unexplored (Koodkaew, 2019; Mankar et al., 2021). We conducted UHPLC analysis using 14 phenylpropanoid compounds in different groups (flavonoids and phenolic acid), which have been reported to be responsible for antioxidant activity in mungbean seeds and sprouts (Kim et al., 2009b; Guo et al., 2012; Gan et al., 2016; Singh et al., 2017). We found that most flavonoid and phenolic acid compounds significantly increased under salinity conditions, including catechin, chlorogenic acid, isovitexin, vitexin, *p*-coumaric acid, syringic acid, and t-ferulic acid (**Table 1**). In buckwheat sprouts, the contents of rutin and vitexin were enhanced under 50 mM NaCl treatment (Lim et al., 2012). The contents of catechin were increased in rice treated with 250 mM NaCl, and chlorogenic, *p*-coumaric, syringic, and t-ferulic acids were also increased by NaCl treatments in the Amaranthus, *brassica* family, rapeseed, and rice (Hassini et al., 2017; Sarker and Oba, 2018; Banik and Bhattacharjee, 2020; Benincasa et al., 2021). In contrast, salinity treatment can decrease some polyphenols in broccoli and romaine lettuce (Kim et al., 2008; López-Berenguer et al., 2009). In rice, while gallic acid, syringic acid, and catechin increased with 250 mM NaCl treatment, myricetin and quercetin decreased (Banik and Bhattacharjee, 2020). We also observed decreased contents of myricetin and quercetin under salinity conditions in the mungbean sprout (**Table 1**). Our findings suggest that the biosynthetic pathways of flavonoids and phenolic acids respond differently to salinity stress, which might be regulated by the key enzymes involved in each pathway.

**Figure 7:**
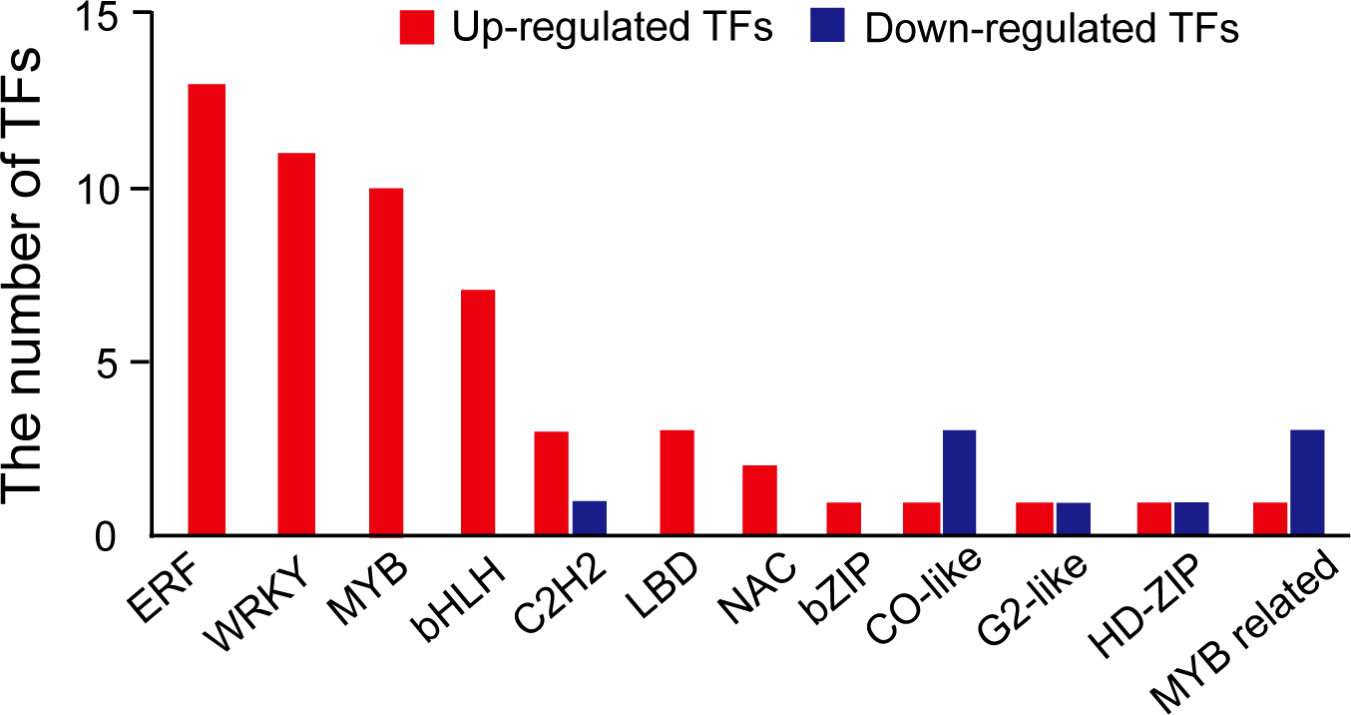
The number of transcription factors identified in differentially expressed genes. Red represents upregulated TF DEGs, and blue represents downregulated TF DEG.

According to the transcriptomic results, some genes (151 downregulated and 528 upregulated) were differentially regulated by NaCl treatment (**Figure 3**). We found that the expression of the genes related to the biosynthesis of the secondary metabolites was significantly increased by NaCl treatment (**Table S2**). *DFR*, *REF1*, *CCOMT*, *COMT*, *HCT*, *CHS*, and *CHI* genes were significantly highly expressed in mungbean sprouts under 50 mM NaCl treatment compared to the control, which corresponded to the changes in the secondary metabolite contents (**Figure 5**). In a previous study, the expression of *TaDFR* and *TaCHI* genes increased following 50 mM NaCl treatment in wheat sprouts (Cuong et al., 2020). In pak choi, *DFR*, *CHS*, and *CHI* genes were upregulated under 250 mM NaCl treatment (Yun et al., 2019). The expression of *CHS* was enhanced in *S. nigrum* as NaCl concentrations increased from 50 to 150 mM (Ben Abdallah et al., 2016). The *CCOMT* and *COMT* genes showed increased expression under salinity in various plants, including basil leaves, barley root, and tomato root (Sugimoto and Takeda, 2009; Manaa et al., 2011; Rastogi et al., 2019). In addition, many plant TF families were identified as upregulated DEGs, including ERFs, WRKYs, and MYBs (**Figure 7**). These TF families often stimulate the metabolic pathway under environmental stress, such as drought and salinity, resulting in more accumulated secondary metabolites in plants (Espley et al., 2007; Ilk et al., 2015; Qiu et al., 2016; Wei et al., 2017; Upadhyay et al., 2018; Li et al., 2019). Our TF DEGs were enriched to “response to stress,” “response to osmotic stress,” “response to salt stress,” “response to abiotic stimulus,” among other responses, and the expressions of these TF DEGs were increased up to log_2_FC 4.5 (log_2_FC 2.0 on average) (**Table S4**). In Arabidopsis, the overexpression of MYB111, MYB1D, and Mdmyb10 TFs significantly increased the expression of *COMT*, *CHS*, and *CHI* genes under salinity stress (Gao et al., 2011; Li et al., 2019). The silencing of OscWRKY1 resulted in reduced expression of *PAL*, *COMT*, and *4CL* transcripts in Arabidopsis (Joshi et al., 2022). In potatoes, *CHS*, *DFR*, and *ANS* genes were upregulated by IbMYB1 overexpression under osmotic stress (Cheng et al., 2013). In this study, four paralogous genes encoding MYB14 were detected as up-regulated DEGs, that had been reported to be positively correlated with key flavonoid synthetic genes, including *ANS*, *ANR*, *DFR*, *CHI*, and *CHS* (Liu et al., 2018). *WRKY72* that had been reported to be associated with the expression of *ANS* and *DFR* was also identified as upregulated TF DEG (Hu et al., 2020). Our transcriptomic results show that the biosynthetic pathways of phenylpropanoids, especially flavonoids, are regulated at the transcription level under salinity stress.

## Conclusion

We found that salinity stress can enhance the accumulation of phenylpropanoids and the expression of their responsive genes in mungbean sprouts. Our results indicate that mild NaCl treatment can be a practical approach to increasing the nutritional value of mungbean sprouts. Our comprehensive metabolomic and transcriptomic data provide insights into the complex regulatory mechanisms for salinity tolerance, as well as valuable information for improving the nutritional quality in legume crops.

## Data availability statement

The data presented in the study are deposited in the NCBI SRA repository, accession number: https://www.ncbi.nlm.nih.gov/bioproject/PRJNA853140.

## Author contributions

IL and MK designed the experiments and wrote the manuscript. IL and MK collected the material data and conducted experiments. BK performed the qualifying and quantifying of the metabolites. IL analyzed the transcriptomic results and contributed to the illustration. JH supervised and revised the manuscript. All authors contributed to the article and approved the submitted version.

## Funding

This work was supported by a grant from the National Research Foundation of Korea (NRF), funded by the Korean government (MSIT; no. 2021R1C1C1004233).

## Conflict of interest

The authors declare that the research was conducted in the absence of any commercial or financial relationships that could be construed as a potential conflict of interest.

## Publisher’s note

All claims expressed in this article are solely those of the authors and do not necessarily represent those of their affiliated organizations, or those of the publisher, the editors and the reviewers. Any product that may be evaluated in this article, or claim that may be made by its manufacturer, is not guaranteed or endorsed by the publisher.
