# Supplementary material for: Metabolomic and transcriptomic changes in mungbean (Vigna radiata (L.) R. Wilczek) sprouts under salinity stress
Source: Front Plant Sci. 2022 Oct 17;13:1030677. doi: 10.3389/fpls.2022.1030677 (PMC9618701; doi:10.3389/fpls.2022.1030677)
Supplement: Supplementary file 1 [file DataSheet_1.docx]

Supplementary Material

# 1. Supplementary Tables

**Supplementary table 1**. Summary information of RNA-seq alignment.

| Sample Name | Raw data | | Clean read | | Aligned (%) |
| --- | --- | --- | --- | --- | --- |
|  | Reads (million) | Base (G) | Reads (million) | Base (G) |  |
| Control-1 | 29.5 | 15 | 28.3 | 14.2 | 98.03 |
| Control-2 | 29.2 | 14.8 | 28.1 | 14.2 | 98.1 |
| Control-3 | 29.0 | 14.8 | 27.9 | 14 | 97.94 |
| Na50-1 | 37.8 | 19.2 | 36.2 | 18.2 | 97.97 |
| Na50-2 | 27.7 | 14 | 36.8 | 13.6 | 98.1 |
| Na50-3 | 37.3 | 19 | 35.9 | 18.1 | 98.34 |

**Supplementary table 2**. List of 10 key differentially expressed genes involved in the phenylpropanoid pathway.

| Gene name | Regulation | Gene ID | Log_2_ Fold Change | *P*-value | FDR | EC number | Description |
| --- | --- | --- | --- | --- | --- | --- | --- |
| DFR | Up | Vradi07g00001339.1 | 1.46 | 4.06242E-05 | 0.001838682 | 1.1.1.219 | Dihydroflavonol 4-reductase |
| REF1 | Up | Vradi02g00003637.1 | 1.86 | 1.35335E-07 | 5.05452E-05 | 1.2.1.68 | coniferyl-aldehyde dehydrogenase |
| REF1 | Up | Vradi02g00003639.1 | 1.01 | 2.95457E-07 | 7.69654E-05 | 1.2.1.68 | coniferyl-aldehyde dehydrogenase |
| CCOMT | Up | Vradi09g00001754.1 | 1.38 | 2.43272E-08 | 1.88456E-05 | 2.1.1.104 | caffeoyl-CoA O-methyltransferase |
| COMT | Up | Vradi03g00001673.1 | 1.76 | 4.47518E-09 | 7.41788E-06 | 2.1.1.68 | catechol O-methyltransferase |
| COMT | Up | Vradi02g00004009.1 | 1.32 | 1.82919E-06 | 0.000228641 | 2.1.1.68 | catechol O-methyltransferase |
| HCT | Up | Vradi09g00002363.1 | 2.36 | 2.1172E-08 | 1.84089E-05 | 2.3.1.133 | shikimate O-hydroxycinnamoyl transferase |
| CHS | Up | Vradi09g00003302.1 | 1.15 | 5.06099E-08 | 3.20174E-05 | 2.3.1.74 | chalcone synthase |
| CHS | Up | Vradi09g00003303.1 | 1.14 | 1.54958E-07 | 5.42749E-05 | 2.3.1.74 | chalcone synthase |
| CHS | Up | Vradi09g00003301.1 | 1.11 | 5.30589E-06 | 0.000459434 | 2.3.1.74 | chalcone synthase |
| CHI | Up | Vradi03g00001100.1 | 1.19 | 6.28204E-05 | 0.002452927 | 5.5.1.6 | chalcone isomerase |

**Supplementary table 3**. List of primer sequence and product size.

| Gene name | Primer sequence | | Product size (bp) |
| --- | --- | --- | --- |
| CCOMT | Forward | ACCTTGCCACAAACAACTCC | 164 |
|  | Reverse | CCATGGTGTTCTTTGCATTG |  |
| CHI | Forward | TTGGAGGATAAAGCGGTGAC | 195 |
|  | Reverse | CAAGTGTGCAACGCAGTTTT |  |
| CHS | Forward | ACTGCAACCCCACCAAACT | 110 |
|  | Reverse | CACATGCGCTGGAACTTCT |  |
| COMT | Forward | ACAGCGAATGAGCTCGGTAT | 113 |
|  | Reverse | GTTGGTGCTTGTGGGTTCTT |  |
| CYP20 | Forward | TCCCCAAACAGCCGAAAA | 107 |
|  | Reverse | CCCCTTGAATCATGAAATCCTT |  |
| DFR | Forward | GGTTCCCACCAAGTTTGAGA | 191 |
|  | Reverse | TATGCACCATGCCATTCACT |  |
| HCT | Forward | CGAGGTTGACCACTTTGGTT | 182 |
|  | Reverse | AAACTCCCCCGTCACTCTTT |  |
| REF1 | Forward | AGCCTGCTGAACAAACACCT | 153 |
|  | Reverse | AACTGACCGCATCAATTTCC |  |
